# Tumor distribution affects bladder recurrence but not survival outcome of multifocal upper tract urothelial carcinoma treated with radical nephroureterectomy

**DOI:** 10.1038/s41598-021-98696-0

**Published:** 2021-09-24

**Authors:** Zai-Lin Sheu, Chi-Ping Huang, Chao-Hsiang Chang, Chung-Hsin Chen, Jian-Hua Hong, Han-Yu Weng, Ta-Yao Tai, Shiu-Dong Chung, Chi-Wen Lo, Thomas Y. Hsueh, Yao-Chou Tsai, Yuan-Hong Jiang, Bing-Juin Chiang, Yung-Tai Chen, Jen-Tai Lin, Wei-Yu Lin, Yeong-Chin Jou, Jen-Shu Tseng, Chia-Chang Wu, Wen‑Jeng Wu, Hsin‑Chih Yeh

**Affiliations:** 1 School of Medicine, College of Medicine, Kaohsiung Medical University, Kaohsiung, Taiwan; 2 Department of Urology, China Medical University and Hospital, Taichung, Taiwan; 3 Department of Urology, National Taiwan University Hospital, College of Medicine, National Taiwan University, Taipei, Taiwan; 4 Institute of Biomedical Engineering, National Taiwan University, Taipei, Taiwan; 5 Department of Urology, National Cheng Kung University Hospital, College of Medicine, National Cheng Kung University, Tainan, Taiwan; 6 Division of Urology, Department of Surgery, Far Eastern Memorial Hospital, Taipei, Taiwan; 7 Division of Urology, Department of Surgery, Taipei Tzu Chi Hospital, The Buddhist Medical Foundation, New Taipei City, Taiwan; 8 Division of Urology, Department of Surgery, Taipei City Hospital Renai Branch, Taipei, Taiwan; 9 Department of Urology, School of Medicine, National Yang-Ming University, Taipei, Taiwan; 10 Department of Urology, Taipei Medical University Hospital, Taipei, Taiwan; 11 Department of Urology, School of Medicine, College of Medicine, Taipei Medical University, Taipei, Taiwan; 12 Department of Urology, Hualien Tzu Chi Hospital, Buddhist Tzu Chi Medical Foundation and Tzu Chi University, Hualien, Taiwan; 13 College of Medicine, Fu-Jen Catholic University, New Taipei City, Taiwan; 14 Department of Urology, Cardinal Tien Hospital, New Taipei City, Taiwan; 15 Department of Life Science, College of Science, National Taiwan Normal University, Taipei, Taiwan; 16 Department of Urology, Taiwan Adventist Hospital, Taipei, Taiwan; 17 Division of Urology, Department of Surgery, Kaohsiung Veterans General Hospital, Kaohsiung, Taiwan; 18 Division of Urology, Department of Surgery, Chang Gung Memorial Hospital, Chia-Yi, Taiwan; 19 Chang Gung University of Science and Technology, Chia-Yi, Taiwan; 20 Department of Medicine, Chang Gung University, Taoyuan, Taiwan; 21 Department of Urology, Ditmanson Medical Foundation, Chiayi Christian Hospital, Chiayi, Taiwan; 22 Department of Health and Nutrition Biotechnology, Asian University, Taichung, Taiwan; 23 Department of Urology, Mackay Memorial Hospital, Taipei, Taiwan; 24 Department of Urology, Shuang Ho Hospital, Taipei Medical University, New Taipei City, Taiwan; 25 TMU Research Center of Urology and Kidney, Taipei Medical University, Taipei, Taiwan; 26 Department of Urology, Kaohsiung Medical University Hospital, No. 100, Shih-Chuan 1st Road, Sanmin Dist., Kaohsiung, 80708 Taiwan; 27 Department of Urology, School of Medicine, College of Medicine, Kaohsiung Medical University, Kaohsiung, Taiwan; 28 Graduate Institute of Medicine, College of Medicine, Kaohsiung Medical University, Kaohsiung, Taiwan; 29 Center for Liquid Biopsy and Cohort Research, Kaohsiung Medical University, Kaohsiung, Taiwan; 30 Department of Urology, Kaohsiung Municipal Ta-Tung Hospital, Kaohsiung, Taiwan

**Keywords:** Urological cancer, Urology

## Abstract

Tumor multifocality and location are prognostic factors for upper tract urothelial carcinoma (UTUC). However, confounding effects can appear when these two factors are analyzed together. Therefore, we aimed to investigate the impact of tumor distribution on the outcomes of multifocal UTUC after radical nephroureterectomy. From the 2780 UTUC patients in the Taiwan UTUC Collaboration Group, 685 UTUC cases with multifocal tumors (defined as more than one tumor lesion in unilateral upper urinary tract) were retrospectively included and divided into three groups: multiple renal pelvic tumors, multiple ureteral tumors, and synchronous renal pelvic and ureteral tumors included 164, 152, and 369 patients, respectively. We found the prevalence of carcinoma in situ was the highest in the synchronous group. In multivariate survival analyses, tumor distribution showed no difference in cancer-specific and disease-free survival, but there was a significant difference in bladder recurrence-free survival. The synchronous group had the highest bladder recurrence rate. In summary, tumor distribution did not influence the cancer-specific outcomes of multifocal UTUC, but synchronous lesions led to a higher rate of bladder recurrence than multiple renal pelvic tumors. We believe that the distribution of tumors reflects the degree of malignant involvement within the urinary tract, but has little significance for survival or disease progression.

## Introduction

Upper tract urothelial carcinoma (UTUC) comprises renal pelvic tumors (RPTs) and ureteral tumors (UTs). It is a relatively rare but aggressive urological cancer with a poor prognosis^1,2^. The current standard treatment for high-risk lesions is radical nephroureterectomy (RNU). However, disease recurrence or metastasis occurs in approximately 20–30% of patients^3–5^. Hence, to provide patients with better management options, a more precise and comprehensive UTUC assessment is needed.

The fact that UTUC spans two neighboring localities has led to the comparison of RPTs and UTs. The incidence of RPTs seem to be higher^3,6,7^ than that of UTs and RPTs often present a more advanced disease stage at the time of diagnosis^6,7^. Conversely, UTs are more likely to be organ-confined and have a greater association with bladder tumor history or recurrence^5,7^. Whether the prognosis of the two tumors is different has therefore been a matter of debate, but recent studies agree that RPTs are associated with better outcomes^3,5,6,8,9^. A possible explanation for this includes the difference in the anatomical nature of RPTs vs UTs. The renal pelvis has tougher physical surroundings that can facilitate a more complete dissection and prevent tumor extrusion, whereas the adventitial layer of the ureter is too thin to be a solid barrier^10^. The differences between RPTs and UTs have been validated, and tumor location is considered a profound factor for the evaluation of UTUC.

In addition to the differences in the disease features of RPTs and UTs, the existence of two localities within a single cancer entity results in heterogeneity of tumor multifocality. Multifocality is not uncommon in UTUC, presenting in 7–42% of patients among different study populations^3,6,11–19^, and leads to worse disease outcomes than single lesions^3,6,8,11–20^. However, the classification of multifocal tumors has failed to reach consistency among various studies^3,6,11,12,14–16,20^. Some definitions of multifocal tumors require the involvement of both the renal pelvis and ureter^3,6,11,16,20^, and some only consider the number of tumors^12,14,15^. Inappropriate grouping may mask the true impact of tumor location and multifocality on prognosis.

With this in mind, we aimed to develop a more explicit classification for multifocal tumors based on tumor distribution. We conducted a retrospective study using the nationwide multi-institution UTUC database to elucidate whether tumor location influences the disease outcomes of patients with multifocal lesions.

## Methods

This nationwide study was conducted by the Taiwan UTUC Collaboration Group and was approved by the Kaohsiung Medical University Hospital Institutional Review Board [KMUHIRB-E(I)-20180214], which waived the need for formal informed consent due to the retrospective nature of the study. All personally identifiable information was removed. The study was performed according to the 1964 Helsinki declaration and strictly followed institutional guidelines and regulations.

A total of 16 participating Taiwanese hospitals contributed to the comprehensive database, which contained data on 2780 patients entered between 1988 and 2020. Exclusion criteria included those who did not receive RNU, those with only a single tumor lesion, and those without complete clinicopathological information. The remaining 685 patients all had multifocal tumors (defined as more than one tumor, regardless of location) and were divided into three groups according to tumor distribution as follows: multiple (defined as more than one tumor in a single anatomical site) RPTs, multiple UTs, and synchronous tumors (RPT + UT). Each group consisted of 164, 152 and 369 patients, respectively.

The database included the following parameters: age, gender, comorbidities [coronary artery disease, end-stage renal disease, hypertension, diabetes mellitus, gout, and non-urothelial carcinoma (UC) malignancy], tumor distribution (multiple or synchronous), history of bladder tumor, pathological tumor grade, pathological tumor stage, lymph node status, histologic variant, carcinoma in situ (CIS), lymphovascular invasion (LVI), tumor necrosis, mortality from UTUC, disease recurrence, and bladder cancer recurrence. Pathological examinations were performed by genitourinary pathologists at each institution based on the same criteria. Tumor staging and grading strictly followed the 2010 American Joint Committee on Cancer TNM classification and the 2004 World Health Organization/International Society of Urologic Pathology consensus classification, respectively.

Regular follow-up appointments were arranged by urologists after RNU. Physical, laboratory and instrumental examinations were all performed according to standard guidelines. Patients with disease recurrence suffered from local relapse in the tumor bed or metastasis to the regional lymph nodes or distant organs; bladder recurrence was regarded as a separate clinical event. The cause of death was determined by attending physicians or death certificates.

For comparison of the demographic and clinicopathological factors of the three groups, Pearson’s chi-square test and Student’s t test were used for categorical and continuous variables, respectively. The Kaplan–Meier method was used to estimate the incidence of disease outcomes, including cancer-specific survival (CSS), disease-free survival (DFS) and bladder-recurrence free survival (BRFS) rates, and the Cox proportional hazards model was selected to evaluate the adjusted survival and recurrence rates. The association of each clinicopathological characteristic with prognosis was examined in the univariate analysis, and those characteristics that showed statistical significance were included in the multivariate analysis. All statistical assessments were two-sided, and p < 0.05 was considered statistically significant. SPSS software, version 26 (IBM; Armonk, NY, USA), was used to perform all analyses.

## Results

Table 1 shows the demographic and clinicopathological features of the three groups based on tumor distribution. There were significant differences in history of bladder tumor (*p* < 0.001), tumor grade (*p* = 0.002), CIS (*p* = 0.028), LVI (*p* = 0.016), and tumor necrosis (*p* = 0.006) among the three groups.

**Table 1:** Demographic and clinicopathological data.

Variables	Multiple RPTs (N = 164)		Multiple UTs (N = 152)		Synchronous RPT and UT (N = 369)		*p* value
	N	%	N	%	N	%	
**Gender**							0.531
Male	75	(45.7)	61	(40.1)	166	(45.0)	
Female	89	(54.3)	91	(59.9)	203	(55.0)	
**Age**							0.712
< 67.7	76	(46.3)	65	(42.8)	172	(46.6)	
≥ 67.7	88	(53.7)	87	(57.3)	197	(53.4)	
**Comorbidity**							
Coronary artery disease	14	(8.5)	11	(7.2)	33	(8.9)	0.816
ESRD on dialysis	27	(16.4)	28	(18.4)	72	(19.5)	0.704
Hypertension	86	(52.4)	81	(53.3)	193	(52.3)	0.979
Diabetes mellitus	40	(24.4)	40	(26.3)	80	(21.7)	0.491
Gout	4	(2.4)	3	(2.0)	16	(4.3)	0.299
Non-UC malignancy	26	(15.9)	20	(13.2)	45	(12.2)	0.516
**History of bladder tumor**							< 0.001**
No	105	(64.0)	70	(46.1)	240	(65.0)	
Yes	59	(36.0)	82	(53.9)	129	(35.0)	
**Tumor grade**							0.002**
Low grade	33	(20.1)	10	(6.6)	46	(12.5)	
High grade	131	(79.9)	142	(93.4)	323	(87.5)	
**Pathological T stage**							0.055
pTa/pTis/pT1	72	(43.9)	64	(42.1)	126	(34.1)	
pT2-4	92	(56.1)	88	(57.9)	243	(65.9)	
**Pathological N stage**							0.738
pN0	45	(27.4)	38	(25.0)	84	(22.8)	
pNx	110	(67.1)	107	(70.4)	261	(70.7)	
pN +	9	(5.5)	7	(4.6)	24	(6.5)	
**Histologic variant**							0.153
No	147	(89.6)	141	(92.8)	321	(87.0)	
Yes	17	(10.4)	11	(7.2)	48	(13.0)	
**Carcinoma in situ**							0.028*
No	139	(84.8)	131	(86.1)	286	(77.5)	
Yes	25	(15.2)	21	(13.8)	83	(22.5)	
**Lymphovascular invasion**							0.016*
No	125	(76.2)	128	(84.2)	267	(72.4)	
Yes	39	(23.8)	24	(15.8)	102	(27.6)	
**Tumor necrosis**							0.006**
No	124	(75.6)	136	(89.5)	303	(82.1)	
Yes	40	(24.4)	16	(10.5)	66	(17.9)	
**Disease recurrence**							0.265
No	115	(70.1)	94	(61.8)	237	(64.2)	
Yes	49	(29.9)	58	(38.2)	132	(35.8)	
**Bladder recurrence**							0.270
No	120	(73.2)	99	(65.1)	249	(67.5)	
Yes	44	(26.8)	53	(34.9)	120	(32.5)	
**Cancer-specific death**							0.880
No	134	(81.7)	125	(82.2)	297	(80.5)	
Yes	30	(18.3)	27	(17.8)	72	(19.5)	

RPTs, renal pelvic tumors; UTs, ureteral tumors; ESRD, end-stage renal disease; UC, urothelial carcinoma.

* < 0.05; ** < 0.01.

### Cancer-specific outcomes

**Figure 1:**
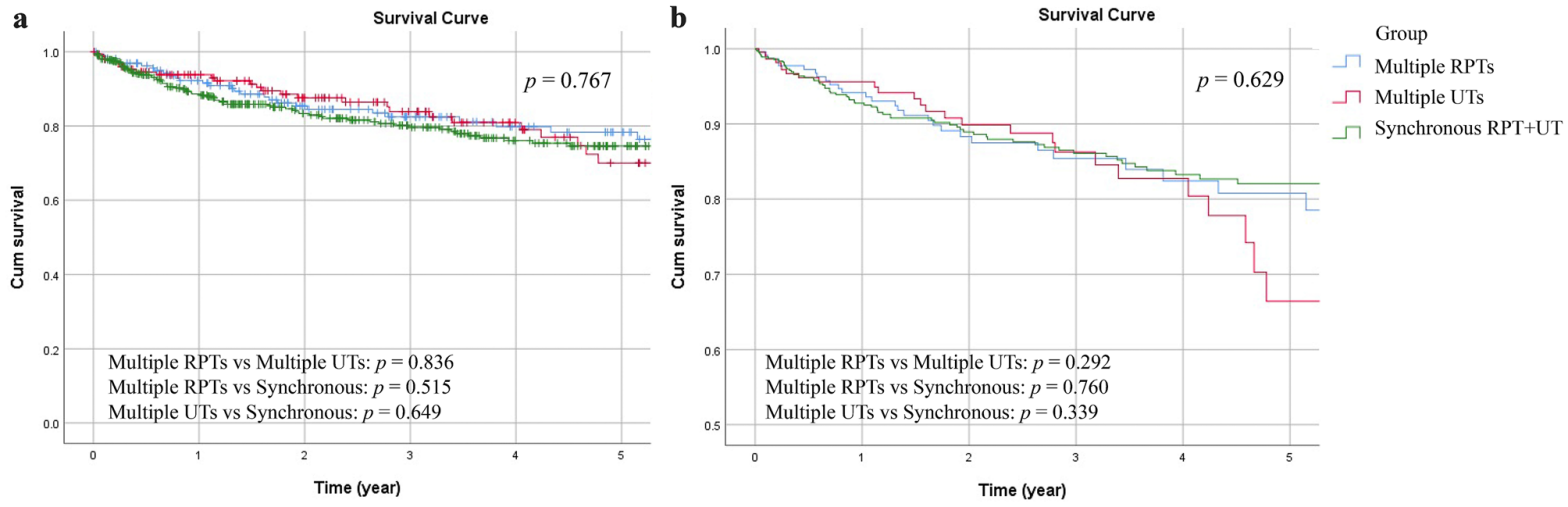
Unadjusted (**a**) and adjusted (**b**) cancer-specific survival (CSS) curves for the three groups.

Cancer-specific outcomes include CSS and DFS. Focusing on patients with multifocal tumors, Table 2 summarizes the univariate and multivariate analyses of the CSS and DFS rates. Regarding the CSS rate, tumor grade (*p* = 0.017), histologic variant (*p* < 0.001)**,** and tumor necrosis (*p* < 0.001) were significant in the univariate analysis but not in the multivariate analysis. Non-UC malignancy (*p* = 0.002; *p* = 0.003), pathological T stage (both *p* < 0.001) and LVI (*p* < 0.001; *p* = 0.002) were significantly associated with CSS in both analyses. The overall 5-year CSS rate in this population was 75%. Figure 1 show the unadjusted and adjusted survival curves of the three groups.

**Table 2:** Comparative univariate and multivariate analyses for cancer-specific outcomes in multifocal UTUC patients.

	Cancer-specific survival				Disease-free survival			
	Univariate analysis		Multivariate analysis		Univariate analysis		Multivariate analysis	
	HR (95% CI)	*p* value	HR (95% CI)	*p* value	HR (95% CI)	*p* value	HR (95% CI)	*p* value
**Gender**		0.278				0.663		
Female	1				1			
Male	1.211 (0.857, 1.710)				1.058 (0.821, 1.365)			
**Age**		0.149				0.648		
< 67.7	1				1			
≥ 67.7	1.293 (0.912, 1.832)				1.061 (0.823, 1.368)			
**Coronary artery disease**		0.642				0.994		
No	1				1			
Yes	1.151 (0.636, 2.086)				0.998 (0.631, 1.578)			
**Hypertension**		0.499				0.724		
No	1				1			
Yes	1.127 (0.797, 1.594)				0.955 (0.741, 1.231)			
**ESRD on dialysis**		0.102				0.003**		0.046*
No	1				1		1	
Yes	0.646 (0.383, 1.091)				0.537 (0.357, 0.807)		0.657 (0.435, 0.993)	
**Diabetes mellitus**		0.636				0.793		
No	1				1			
Yes	1.100 (0.741, 1.634)				0.960 (0.711, 1.298)			
**Gout**		0.170				0.510		
No	1				1			
Yes	0.376 (0.093, 1.521)				0.788 (0.389, 1.598)			
**Non-UC malignancy**		0.002**		0.003**		0.375		
No	1		1		1			
Yes	1.973 (1.272, 3.061)		1.974 (1.261, 3.090)		1.184 (0.815, 1.720)			
**History of bladder tumor**		0.156				0.306		
No	1				1			
Yes	1.288 (0.908, 1.828)				1.144 (0.884, 1.481)			
**Tumor grade**		0.017*		0.489		< 0.001**		0.058
Low grade	1		1		1		1	
High grade	2.197 (1.152, 4.191)		0.780 (0.386, 1.576)		3.374 (1.929, 5.904)		1.765 (0.980, 3.176)	
**Pathological T stage**		< 0.001**		< 0.001**		< 0.001**		< 0.001**
pTa/pTis/pT1	1		1		1		1	
pT2-4	5.422 (3.211, 9.158)		4.397 (2.473, 7.818)		3.736 (2.691, 5.187)		2.539 (1.776, 3.630)	
**Pathological N stage**		0.176		0.638		0.001**		0.268
pN0	1		1		1		1	
pNx	0.741 (0.493, 1.113)	0.149	0.670 (0.444, 1.009)	0.055	0.996 (0.726, 1.365)	0.979	0.899 (0.654, 1.236)	0.512
pN +	2.644 (1.471, 4.753)	0.001**	1.120 (0.600, 2.091)	0.723	3.590 (2.273, 5.672)	< 0.001**	1.594 (0.976, 2.603)	0.063
**Histologic variant**		< 0.001**		0.400		< 0.001**		0.548
No	1		1		1		1	
Yes	2.251 (1.454, 3.485)		1.226 (0.762, 1.972)		2.027 (1.437, 2.859)		1.122 (0.771, 1.631)	
**Carcinoma in situ**		0.811				0.684		
No	1				1			
Yes	0.947 (0.603, 1.486)				0.933 (0.669, 1.302)			
**Lymphovascular invasion**		< 0.001**		0.002**		< 0.001**		0.001**
No	1		1		1		1	
Yes	2.979 (2.099, 4.230)		1.848 (1.264, 2.701)		2.626 (2.020, 3.415)		1.610 (1.203, 2.154)	
**Tumor necrosis**		< 0.001**		0.123		< 0.001**		0.040*
No	1		1		1		1	
Yes	2.125 (1.459, 3.094)		1.382 (0.916, 2.087)		1.934 (1.448, 2.583)		1.387 (1.015, 1.896)	
**Tumor distribution**		0.477		0.988		0.136		0.622
Multiple RPTs	1		1		1		1	
Multiple UTs	1.042 (0.619, 1.752)	0.878	1.334 (0.780, 2.280)	0.292	1.324 (0.905, 1.937)	0.148	1.404 (0.953, 2.069)	0.086
Synchronous RPT and UT	1.157 (0.755, 1.771)	0.503	1.069 (0.695, 1.646)	0.760	1.314 (0.946, 1.824)	0.103	1.145 (0.823, 1.592)	0.421

RPTs, renal pelvic tumors; UTs, ureteral tumors; ESRD, end-stage renal disease; UC, urothelial carcinoma; CI, confidence; HR, hazard ratio.

* < 0.05; ** < 0.01.

**Figure 2:**
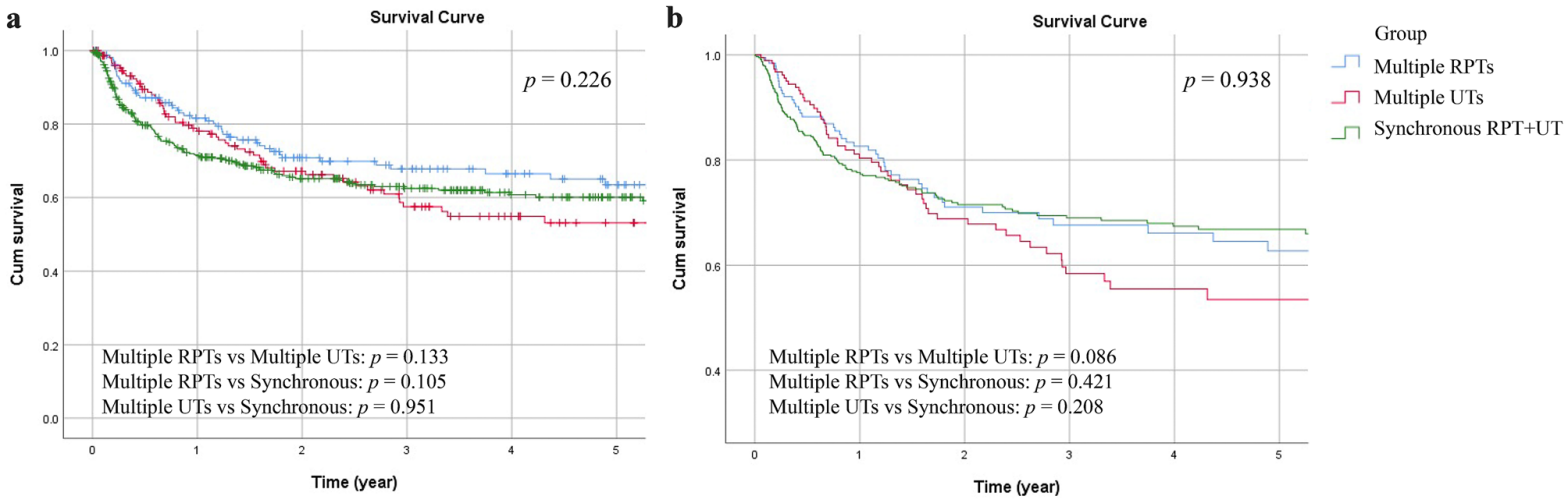
Unadjusted (**a**) and adjusted (**b**) disease-free survival (DFS) curves for the three groups.

For the DFS rate, tumor grade (*p* < 0.001), pathological N stage (*p* = 0.001) and histologic variant (*p* < 0.001) were only significant in the univariate analysis, while end-stage renal disease on dialysis (*p* = 0.003; *p* = 0.046), pathological T stage (both *p* < 0.001), LVI (*p* < 0.001; *p* = 0.001), and tumor necrosis (*p* < 0.001; *p* < 0.04) were demonstrated to be independent prognostic factors for the DFS rate in both analyses (Table 2). Overall, the 5-year DFS rate was 60%, and the corresponding DFS rate curve is shown in Fig. 2.

### Bladder recurrence

**Figure 3:**
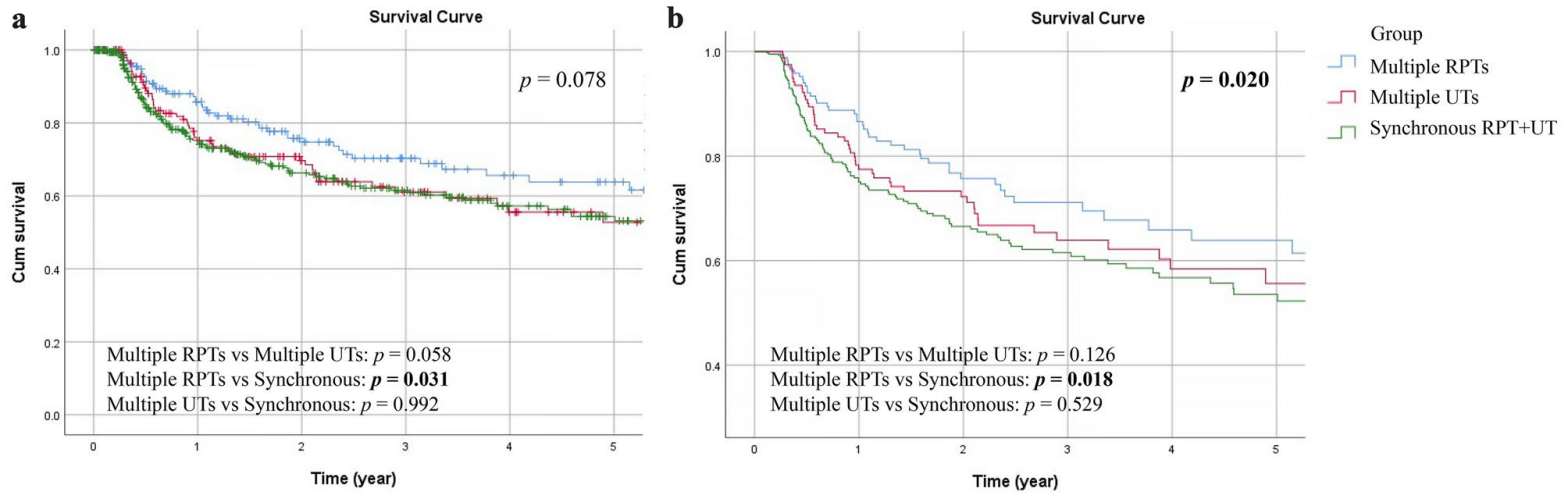
Unadjusted (**a**) and adjusted (**b**) bladder recurrence-free survival (BRFS) curves for the three groups.

The overall 5-year BRFS rate in this cohort was 57%. In the univariate analysis, age (*p* = 0.014), gender (*p* = 0.001), gout (*p* < 0.001), history of bladder tumor (*p* = 0.002), non-UC malignancy (*p* = 0.022), and tumor distribution (*p* = 0.046) were significantly correlated with the BRFS rate (Table 3). Additionally, age (*p* = 0.014), gender (*p* = 0.020), gout (*p* < 0.001), history of bladder tumor (*p* = 0.002), and tumor distribution (*p* = 0.020) showed significant associations with the BRFS rate in the multivariate analysis (Table 3). Figure 3 demonstrates the cumulative BRFS of the three groups, and the adjusted curve shows that the recurrence rate of bladder cancer in the synchronous group is higher than that in the multiple RPT group (Fig. 3b, *p* = 0.018).

**Table 3:** Comparative univariate and multivariate analyses for bladder-recurrence free survival in multifocal UTUC patients.

	Univariate analysis		Multivariate analysis	
	HR (95% CI)	*p* value	HR (95% CI)	*p* value
**Age**		0.014*		0.014*
< 67.7	1		1	
≥ 67.7	1.400 (1.070, 1.832)		1.405 (1.071, 1.843)	
**Gender**		0.001**		0.020*
Female	1		1	
Male	1.593 (1.219, 2.081)		1.387 (1.053, 1.826)	
**Coronary artery disease**		0.401		
No	1			
Yes	1.218 (0.769, 1.930)			
**Hypertension**		0.191		
No	1			
Yes	1.195 (0.915, 1.561)			
**ESRD on dialysis**		0.581		
No	1			
Yes	0.904 (0.632, 1.293)			
**Diabetes mellitus**		0.258		
No	1			
Yes	1.192 (0.879, 1.616)			
**Gout**		< 0.001**		< 0.001**
No	1		1	
Yes	3.082 (1.849, 5.136)		3.107 (1.827, 5.284)	
**Non-UC malignancy**		0.022*		0.039*
No	1		1	
Yes	1.565 (1.068, 2.294)		1.507 (1.022, 2.222)	
**History of bladder tumor**		0.002**		0.002**
No	1		1	
Yes	1.531 (1.172, 2.000)		1.571 (1.182, 2.088)	
**Tumor grade**		0.601		
Low grade	1			
High grade	1.109 (0.754, 1.631)			
**Pathological T stage**		0.508		
pTa/pTis/pT1	1			
pT2-4	1.096 (0.836, 1.437)			
**Pathological N stage**		0.963		
pN0	1			
pNx	1.068 (0.771, 1.478)	0.694		
pN +	0.879 (0.431, 1.794)	0.723		
**Histologic variant**		0.108		
No	1			
Yes	0.651 (0.385, 1.100)			
**Carcinoma in situ**		0.811		
No	1			
Yes	1.042 (0.742, 1.465)			
**Lymphovascular invasion**		0.381		
No	1			
Yes	1.153 (0.838, 1.587)			
**Tumor necrosis**		0.896		
No	1			
Yes	1.024 (0.719, 1.459)			
**Tumor distribution**		0.046*		0.020*
Multiple RPTs	1		1	
Multiple UTs	1.460 (0.979, 2.177)	0.064	1.371 (0.916, 2.053)	0.126
Synchronous RPT and UT	1.461 (1.034, 2.064)	0.032*	1.526 (1.077, 2.164)	0.018*

RPTs, renal pelvic tumors; UTs, ureteral tumors; ESRD, end-stage renal disease; UC, urothelial carcinoma; CI, confidence; HR, hazard ratio.

* < 0.05; ** < 0.01.

## Discussion

Previous studies showed that multifocal tumors had a worse prognosis and higher progression rate than single lesions^12–14,19^. A possible explanation included the correlation of multifocal tumors with a larger volume of disease^20^. Moreover, 61.8% of the patients with multifocal tumors in this population presented with pT2 stage disease or higher at the time of diagnosis. This result was similar to that of the study by Chromecki et al.^12^, in which multifocal tumors were found to be more advanced than single lesions (65.1% vs 50.6% of tumors at stage pT2 or higher). Such extensive involvement and aggressive tumor behavior are likely to worsen patient prognosis. In addition, a correlation between the total tumor load and tendency of metastasis has been observed in other cancers^21^. We hypothesize that a similar trend might exist in UTUC, and this requires further research.

However, UTUC arises from two anatomical sites that present different biological contextures, and tumor location has been shown to have prognostic significance in recent studies^3,6,8,9^. It is unknown whether the distribution of lesions has an impact on the disease outcomes of multifocal UTUC. In previous analyses, the definitions of tumor multifocality were dissimilar. We reviewed the literature and found that tumor multifocality, tumor multiplicity, and synchronous RPT and UT were individually reported to be correlated with poor prognosis^3–6,11–20^. However, comparisons between multiple and synchronous tumors are lacking. Therefore, we divided patients with multifocal tumors into three groups according to tumor distribution for outcome analysis.

Under strict patient selection, we first sought to clarify whether the cancer-specific outcomes of multifocal tumors were affected by tumor distribution. We compared multiple RPTs, multiple UTs and synchronous tumors and found no difference in the CSS and DFS rates between the groups. Thus, we considered that tumor multifocality as a whole may exert a dominant impact on cancer-specific outcomes regardless of how the tumors are distributed. Moreover, the development of multiple lesions might also blur the distinction of localities, since multiple lesions in one location may encroach on the other location. Therefore, differences in the CSS and DFS rates among the three groups may have been obscured.

Although tumor distribution seemed to have little impact on cancer progression and prognosis in patients with multifocal tumors, our study did find differences in bladder recurrence rates based on tumor distribution in the multivariate analysis. Previous studies appeared to indicate that the bladder recurrence rate was higher in multifocal tumors compared to single tumors^14,15,18,19^. Through the subdivision of multifocal tumors, our analysis showed that the bladder recurrence rate of synchronous tumors was significantly higher than that of multiple RPTs. The results of Fradet et al.^17^ and our previous study^5^ partially supported our findings by demonstrating no significant difference in the BRFS rate between patients with multiple lesions and those with single lesions in multivariate analyses. In summary, we believe that within multifocal tumors, synchronous lesions have a more profound impact on intravesical recurrence compared with multiple RPTs.

There are two main theories for the development of multifocal UTUC. The first is field cancerization, in which carcinogens cause independent genetic alterations in different sites of the urothelium^22^. Although all included patients had multifocal lesions, the urinary tract involvement in patients with synchronous tumors appeared to be more extensive than in patients with multiple RPTs because they had skip lesions affecting the renal pelvis and ureter. The hypothesis is also supported by the higher incidence of urothelial CIS in synchronous tumors (Table 1, *p* = 0.028), because CIS is usually multifocal and can be a manifestation of extensive filed cancerization. Therefore, we consider that patients with synchronous tumors may have a wider range of cancerous changes, which are more likely to further develop bladder recurrence.

Tumor seeding is another hypothesis of multifocal UTUC, indicating that monoclonal cancer cells from the first tumor flow antegrade and redeposit in the urinary tract to grow new lesions^23,24^. We assumed that patients with multiple RPTs may be less likely to experience remote seeding compared to patients with synchronous tumors characterized by long-distance spread (renal pelvis to ureter). Therefore, patients with synchronous tumors had a higher risk of recurrence at the distal end (bladder).

While comprehensive molecular or genetic evidence is still needed, the findings of this study have clinical relevance. A single dose of intravesical chemotherapy after RNU has been suggested for high-risk patients by the authors of several studies and in the latest guideline to prevent bladder cancer development^2,25,26^. Our results indicated that those with synchronous tumors had the highest risk of bladder recurrence and may benefit from multiple intravesical instillations with stricter follow-up regimens.

Another finding of our study was the association of gout with bladder cancer recurrence in patients with UTUC. Previous studies have shown a higher risk of cancer development in patients with gout, especially the development of bladder cancer^27,28^. Based on our analysis, patients with gout were three times more likely to experience intravesical recurrence after RNU. Therefore, we suggest that these patients should also be closely monitored after RNU and prophylactic intravesical therapy.

Several limitations existed in this study because of its retrospective nature. First, among the multifocal tumors, only the pathological features of the dominant lesion were collected, preventing detailed analyses of individual lesions. Additionally, a centralized pathological review was difficult to conduct because this was a multi-institutional study. Furthermore, surgical practice may have changed over time throughout the three decades and, therefore, could have biased the disease outcomes. Nonetheless, this study provided insight into tumor multifocality and identified that tumor distribution had limited association with cancer-specific outcomes; however, the specific population with synchronous tumors had an increased risk for bladder recurrence.

In conclusion, multifocal UTUC represents a heterogeneous population in which tumor distribution affects disease outcomes to varying degrees. We demonstrated no difference in the CSS and DFS rates among patients with multiple RPTs, multiple UTs and synchronous tumors. However, synchronous tumors portended a higher bladder recurrence rate than multiple RPTs, which should be considered in clinical assessment. We believe that synchronous tumors may be a manifestation of widespread malignant involvement of the entire urinary tract, but have limited significance in the cancer-specific outcomes of multifocal UTUC.

## Data Availability

The authors declare that all data analyzed during the current study are available from the corresponding author upon reasonable request.
